# Efficacy of idebenone in the Treatment of iRBD into Synucleinopathies (EITRS): rationale, design, and methodology of a randomized, double-blind, multi-center clinical study

**DOI:** 10.3389/fneur.2022.981249

**Published:** 2022-09-12

**Authors:** Yuanyuan Li, Chunyi Wang, Ningdi Luo, Fangzheng Chen, Liche Zhou, Mengyue Niu, Wenyan Kang, Jun Liu

**Affiliations:** ^1^Department of Neurology and Institute of Neurology, Ruijin Hospital Affiliated With Shanghai Jiaotong University School of Medicine, Shanghai, China; ^2^CAS Center for Excellence in Brain Science and Intelligence Technology, Ruijin Hospital Affiliated With Shanghai Jiaotong University School of Medicine, Shanghai, China

**Keywords:** idebenone, rapid eye movement sleep behavior disorder (RBD), α-synucleinopathies, efficacy and safety, randomized controlled trial

## Abstract

**Background:**

As the strongest prodromal marker of α-synuclein-specific neurodegeneration, idiopathic REM sleep behavior disorder (iRBD) is becoming a focus of interest in disease-modifying therapy. Idebenone has been widely portrayed as a potent antioxidant targeting mitochondrial dysfunction. Previous study has identified the effect of idebenone on Parkinson's disease with promising outcomes by regulating mitophagy. A novel indication of idebenone should be highlighted in iRBD population.

**Methods:**

The EITRS study is a randomized, double-blind, multi-center clinical study assessing the efficacy and safety of idebenone in the treatment of iRBD into synucleinopathies. One hundred forty-two patients (aged 40–75 years old) with clinically diagnosed iRBD are planned to be recruited with 80% statistical power and randomly assigned to idebenone (30 mg each time, three times a day) or matching placebo orally for 5 years. The assessment of rating scales, blood testing and neuroimaging examinations will be conducted at baseline, the 1st, 3rd and 5th year of follow-up. The primary efficacy endpoint is the 5-year conversion rate in patients with iRBD. The secondary endpoint is the safety and tolerability of idebenone in the treatment of iRBD. The study has been launched in July 2020.

**Discussion:**

This is the first prospective study designed to identify the efficacy and safety of idebenone on the treatment of iRBD into synucleinopathies. The current results are expected to promote the development of evidence-based recommendations for the management of patients with iRBD. Furthermore, we hope to provide insights on a possible disease-modifying approach with robust evidence.

**Trial Registration:**

Clinicaltrials.gov, identifier: NCT 04534023.

## Background

Rapid eye movement (REM) sleep behavior disorder (RBD) is a parasomnia characterized by dream enactment behaviors and the loss of physiological muscle atonia during REM sleep (1). RBD has been categorized into idiopathic RBD (iRBD) and symptomatic forms, the latter of which is acknowledged as a precursory sign of α-synucleinopathies, including Parkinson's disease (PD), dementia with Lewy bodies (DLB), and multiple system atrophy (MSA), etc. (2). α-synucleinopathies are a group of neurodegenerative disorders characterized by aggregates of α-synuclein in neurons and glia, in the form of Lewy bodies, Lewy neurites, neuronal cytoplasmic inclusions, and glial cytoplasmic inclusions (3). More than 80–90% of iRBD patients ultimately develop a clinically defined neurodegenerative disease over longitudinal follow-up (4). Consequently, iRBD is considered as a recognizable preclinical state to enable the identification of individuals at risk of neurodegeneration (5). However, the pathogenesis and the potential mechanism still need investigation with little understanding of the reliable biomarkers of phenoconversion, which hinders the early diagnosis and intervention. Recent animal models and brain imaging researches revealed the degeneration of the core brain stem circuits controlling REM sleep in RBD pathology (6, 7). The initial REM sleep atonia loss (RSWA) and RBD symptoms accord with the pathological progression of Lewy body in PD which begins in the medulla and pons (8–10). It is speculated that α-synuclein could begin in the caudal brain stem affecting REM sleep circuits and progressively propagate to substantia nigra and other brain region (11). Besides, iRBD patients frequently exhibit specific neurodegenerative biomarkers, including: (1) clinical symptoms of hyposmia, constipation, orthostatic hypotension, cognitive decline, and gait initiation abnormalities; (2) structural or functional magnetic resonance imaging (MRI) signifying decreased nigrostriatal putamenal dopaminergic uptake, altered neuromelanin signal intensity and cortical thinning, as well as the brain glucose metabolism measured by ^18^F-FDG PET positron emission tomography (^1^8F-FDG PET) and dopamine transporter deficit by ^123^I-N-ω-fluoropropyl-2β-carbomethoxy-3β-(4-iodophenyl) nortropane single-photon emission computed tomography (^123^I-FP-CIT SPECT); (3) neurophysiological markers such as electroencephalographic slowing, etc (5, 12, 13). The signs may be important predictors for impendent phenoconversion, while the application should be integrated considering higher sensitivity and specificity.

The management of iRBD is principally focused on physical protection and pharmacotherapy (1, 14). Clonazepam and melatonin are widely prescribed in the clinical practice as grade B recommendation (15). Clonazepam can significantly reduce the occurrence of RBD behavior and trauma, whereas it should be applied with caution on patients with dementia, gait abnormalities or obstructive sleep apnea syndrome (16). The administration should also be monitored strictly especially on patients with neurodegenerative diseases or abnormal liver function. Long-term application may contribute to excessive daytime sedation, movement disorders, blurred consciousness, amnesia, etc (1). Instead, melatonin has a much safer profile with fewer and milder side effects. Its efficacy has been corroborated in the treatment of iRBD with or without concurrent DLB, PD or MSA (17, 18). The dose-related adverse reactions of melatonin most commonly include morning headache, daytime sleepiness, delusions and hallucinations (17). Nonetheless, evidence of both the medications above is still insufficient to validate their utility on phenoconversion in the prodromal stage.

Mitochondrial dysfunction has been verified predominately responsible for dopaminergic neuron degeneration, which can be promising as a novel therapeutic target (19). Idebenone is a potent antioxidant and inhibitor of lipid peroxidation that is capable of stimulating mitochondrial electron flux and cellular energy production, which ensures the integrity of mitochondrial function and structure (20). Idebenone may exert a protective effect against neuroinflammation and microglial activation in PD. Complementary therapy with idebenone addressed motor and non-motor issues and mitigated the pathology of PD in animal and cell models (21–25). However, the extensive mechanisms need better interpretation and solid evidence is warranted to quantify the effectiveness and safety in clinical practice. Herein, we describe the design and rationale of a prospective clinical trial to identify whether idebenone can reduce the conversion rate or postpone the onset of neurodegeneration effectively in the treatment of iRBD.

## Methods

### Study aims

The primary aim of the study is to investigate the 5-year conversion rate of α-synucleinopathies in iRBD patients treated with idebenone. The secondary aim is to clarify the safety and tolerability of idebenone in the treatment of iRBD patients over longitudinal follow-up.

### Study designs

**Figure 1:**
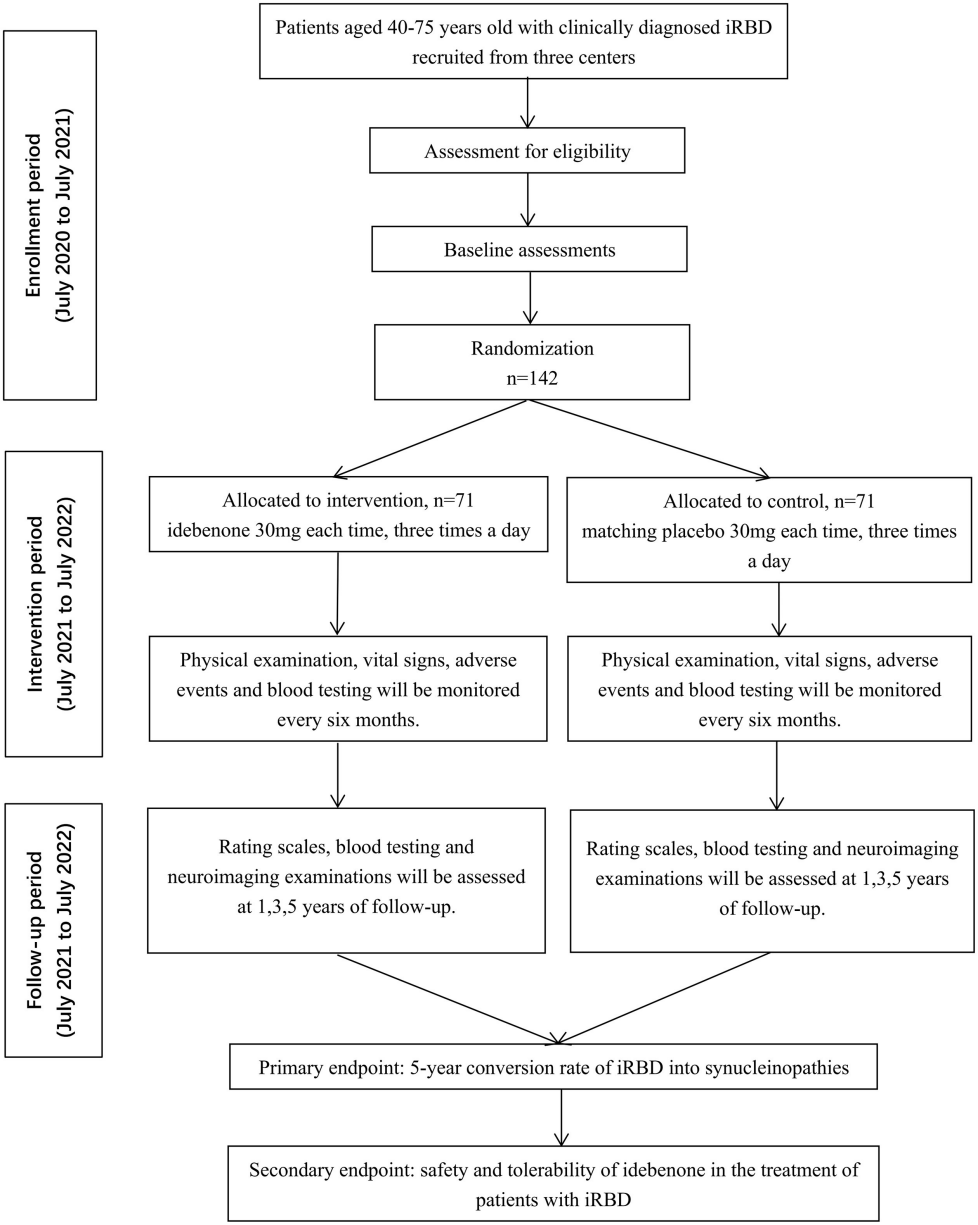
Flowchart of study design.

EITRS is a randomized, double-blind, multi-center clinical study with two parallel arms in 1:1 ratio to evaluate the efficacy and safety of idebenone in patients with iRBD compared with placebo. The total study duration of 6 years comprises a 1-year enrollment period and a 5-year intervention period. One hundred forty-two eligible participants will be allocated to idebenone or placebo group randomly and equally after baseline assessment. Follow-up evaluations will be conducted at the 1st, 3rd and 5th years of treatment in any of the three centers to detect the disease progression. Routine blood testing and liver function will be monitored every 6 months during the administration. Participants, investigators and statisticians are masked to the allocation code. The flowchart of the trial is outlined in Figure 1. The EITRS study is registered with ClinicalTrial.gov (Register Number: NCT 04534023).

### Patients and study sites

In total, 142 outpatients were recruited from the department of Neurology of three centers in Shanghai, Suzhou and Wuhan: Ruijin Hospital affiliated to Shanghai Jiaotong University School of Medicine, the Second Affiliated Hospital of Soochow University and Union Hospital, Tongji Medical College, Huazhong University of Science and Technology. Among them, Ruijin Hospital is the leader of EITRS, in charge of patient enrollment, quality control and ethical problems. Patients were enrolled between July 2020 (study start date) and July 2021; the study end date (last patient completed the study) was July 2026. The inclusion and exclusion criteria are depicted as follows.

### Inclusion criteria

1. Male or female, aged 40–75;
2. The diagnosis of RBD was confirmed by polysomnography (PSG) according to standard criteria published by the American Academy of Sleep Medicine in the International Classification of Sleep Disorders (ICSD) III (26);
3. Patients voluntarily participated in this study and signed informed consent with good compliance.

### Exclusion criteria

1. PSG-confirmed sleep apnea hypopnea syndrome, which includes but not limited to obstructive sleep apnea syndrome (apnea/hypopnea index >5) (26);
2. Patients with symptoms including bradykinesia, myotonia, tremor or posture instability;
3. Previous history of sudden withdrawal of sedative or psychotropic drugs prior to the onset of the symptoms;
4. Previous history of neurological diseases such as cerebral hemorrhage, cerebral infarction, brain trauma, brain tumor or central nervous system infection;
5. Previous history of sleep disorders or seizure;
6. Alcohol or drug dependence (addiction).

### Endpoint criteria

1. Patients finish 5-year idebenone treatment;
2. Patients develop overt α-synucleinopathies according to the diagnostic criteria. The definition of α-synucleinopathies in the endpoint contains three most common types: PD, DLB and MSA.

### Discontinue criteria of intervention

1. Severe liver and kidney function abnormality associated with the intervention (transaminase increase more than twice of the normal value);
2. Severe blood testing abnormality: red blood cell, hemoglobin, white blood cell or platelet count <50% of the lower normal limit, or more than twice the upper normal limit;
3. Severe allergic reactions or arrhythmias: severe hypertension (blood pressure > 220/120 mmHg) or hypotension (blood pressure < 80/60 mmHg) caused by intervention; or severe arrhythmias (heart rate < 50 beats/min, heart rate > 140 beats/min or atrial fibrillation) and sinus arrest; or respiratory disorder (oxygen saturation < 85%);
4. Consciousness disorders: coma, delirium or convulsions.

### Discontinue/withdraw criteria of patients

1. Pregnant;
2. Poor compliance to the protocol;
3. Serious complications or other severe diseases happen during the administration and the continuation shows little benefits;
4. Significant disease progression and the discontinuation is necessary after evaluation;
5. Meet the exclusion criteria (newly or already confirmed);
6. Unable to receive the intervention as scheduled.

Participants will be included in the final statistical analysis if they accomplish three courses of treatment and evaluation, otherwise will be excluded.

### Recruitment, screening and baseline assessment

Patients from the three centers will be recruited successively from July 2020 to July 2021. The baseline information will be collected and physical examinations will be conducted to assess the eligibility of each patient. Baseline demographics include sex, age, education level, occupation, concomitant disease and medications, etc. Blood testing is required to detect the blood biomarkers (neuronal exosomal α-synuclein, SNCA methylation, inflammatory cytokines and chemokines), as well as liver and kidney function to exclude patients with contraindications. In addition, patients will undergo (^18^F-FP-CIT) dopamine transporter (DAT)-PET/ MRI neuroimaging examinations.

### Randomization and blinding procedures

A random-number table based on block randomization was prepared by an independent biostatistician. Patients will be randomized equally to idebenone or identical-looking placebo group. If eligible individuals are invited to participate in the study and provide written informed consent, the randomized code and treatment will be assigned in a sealed envelope. Assessors are specific in each center, and both investigators and patients will remain fully blinded to the allocation throughout the study.

### Intervention and follow-up

During the 5-year treatment, patients will undergo oral administration of idebenone or placebo with a dosage of 30 mg each time, three times a day. Given idebenone is prescribed conventionally for cerebral infarction, cerebral hemorrhage, and atherosclerosis sequelae caused by brain dysfunction, the optimal dosage is recommended by the instruction of idebenone (30 mg per tablet, Shenwei, Qilu Pharmaceuticals, China). We also deliberated the administration according to preliminary literatures and clinical trials in the use of neurodegenerative disorders (27–29). Therefore, the dosage is fixed without setting dose gradient as control groups. The drug will be kept by blinded nurses, and distributed to patients according to the code. Routine blood testing and liver function will be monitored every 6 months during the administration. Once serious side effects occur, such as leukopenia, liver function damage or severe allergic reaction, participants should discontinue or withdraw from the trial. The follow-up evaluations of patients include the assessment of rating scales, blood testing and neuroimaging examinations. Movement Disorder Society-Unified Parkinson's Disease Rating Scale (MDS-UPDRS), Mini-Mental State Examination (MMSE), Montreal Cognitive Assessment (MoCA), Sniffin' Sticks test-16 (SS-16), 17-Item Hamilton Rating Scale for Depression (HAMD-17), Non-Motor Symptom Questionnaire (NMSQ) and Scales for Outcomes in Parkinson's Disease-Autonomic questionnaire (SCOPA-AUT) is used to quantify the progression of motor and non-motor symptoms. In view of the favorable improvement on neurodegenerative disease of idebenone, it is speculated that idebenone might ameliorate RBD symptoms as well (30). Therefore, we use REM sleep behavior disorder screening questionnaire (RBDSQ) to identify the influence. Plasma exosome-synuclein levels will be tested to provide convictive biomarkers for neurodegeneration. Several inflammatory cytokines and chemokines (interleukin-6 (IL-6), IL-8, IL-10, tumor necrosis factor-α (TNF-α), TGF-β, C-X-C motif ligand (CXCL) 12, C-X3-C motif ligand 1 (CX3CL1), C-C motif ligand (CCL) 3, CCL15 and CCL20) will be measured based on our previous investigation (31). The SNCA methylation will also be analyzed by bisulfite sequencing (32, 33). In addition, according to our previous publications, functional connectivity impairment and increased free water in the posterior substantia nigra are indicative imaging markers for phenoconversion (34–36). PET/MRI can also provide supplementary evidence for dopaminergic neurodegeneration, which is necessary for the participants in the follow-up evaluation.

### Outcome measures

The primary outcome is the comparison of conversion rate from iRBD to synucleinopathies between idebenone and placebo group, measured at 5-year of follow-up. Clinical symptoms will be evaluated by physicians using comprehensive rating scales. PET/MRI will be carried out to reflect the dopaminergic degeneration. The secondary outcome is the safety and tolerability of idebenone in the treatment of patients with iRBD. The objects for safety evaluation should contain all the participants that have been administrated with the experimental drug. Safety evaluation including the laboratory test results, the events of death and adverse reactions, will be recorded in detail and analyzed after the trial (Table 1).

**Table 1:** Outcome measures taken at baseline and follow-up.

**Endpoint**		**Measure**	**Evaluating methods/criterion**	**Time points**
Primary	Effectiveness: 5-year conversion rate of iRBD into synucleinopathies	Rating scales	MDS-UPDRS, RBDSQ, MMSE, MoCA, SS-16, HAMD-17, NMSQ, SCOPA-AUT	Baseline, 1, 3, 5 years
		Blood testing	Biomarkers including neuronal exosomal α-synuclein, SNCA methylation, inflammatory cytokines and chemokines (IL-8, TNF-α, TGF-β, IL-10, IL-6, CXCL12, CX3CL1, CCL15, CCL3 and CCL20)	
		Neuroimaging examinations	(^18^F-FP-CIT) DAT-PET/ MRI	
Secondary	Safety and tolerability	Vital signs	Body temperature > 39.5°C, pulse rate <50 beats/min, blood pressure < 80/60 mmHg, blood pressure > 220/120 mmHg, oxygen saturation < 90%;	Every 6 months
		Electrocardiogram	Atrial fibrillation, complete atrioventricular block, sinus arrest, ventricular tachycardia, ventricular fibrillation, pathologic Q-waves, arched ST-segment elevation	
		Laboratory tests	The ratio of alanine aminotransferase to aspartate aminotransferase over twice the normal values after treatment, the creatinine over twice the normal value, glomerular filtration rate lower than 20 ml/min, red blood cell, hemoglobin, white blood cell and platelet count <50% of the lower normal limit or more than twice the upper normal limit	
		Adverse events	Result in death, are immediately life-threatening or cause severe organ damage	

### Serious adverse event and adverse event reporting

An AE is defined as any untoward medical occurrence in the patients which does not necessarily have a causal relationship with this treatment. These are usually identified by the physicians during the follow-up.

A SAE is an AE that fulfills one or more of the following criteria:

1. Results in death;
2. Is immediately life-threatening;
3. Causes severe organ damage.

The baseline information including vital signs, previous medical history, routine blood testing, liver and kidney function, will be recorded in detail before the intervention. The index will be monitored and compared with the baseline periodically during the follow-up. Complaints from participants will be carefully recorded and physical examinations should be conducted if necessary.

Once the AE or SAE is identified, the therapeutic regimen will be adjusted accordingly. Description of the detailed event, time of occurrence, professional assessment of the severity, relationship with the study intervention, and time from occurrence to resolution/stabilization should be recorded in the case report form, which will also be reported to the principal investigator, hospital ethics committee and medical affair department. The detailed reasons will be explained to patients responsibly and the subsequent solution will be suggested from a professional perspective. Patients should be followed up until proper resolution.

### Sample size and statistical analysis

The 5-year conversion rate of participants treated with idebenone is expected to decrease from 47% in the placebo group to 25%. The idebenone and placebo group are randomized at a ratio of 1:1, and a 5% drop-out rate is considered throughout the study. We estimated that 71 patients would be needed in each treatment group to allow for a power of 80% at two-sided α-level of 5%. In total, 142 patients are projected to be enrolled.

Statistical analysis will be performed using SPSS 27.0, STATA 17.0 and GraphPad Prism 9.4.0. Data will be analyzed on an intention-to-treat basis using two-sided tests with *p* < 0.05 considered significant. Continuous variables will undergo normality test as well as ANOVA test for inter-group comparison. Categorical variables will be compared using Chi-square test, Fisher's exact test or Wilcoxon rank-sum test. To assess the five-year conversion rate from iRBD diagnosis to clinically diagnosed synucleinopathies between patients treated with idebenone and placebo, we estimated the hazard ratio (HR) using a Cox proportional hazards model. Kaplan-Meier method will be applied to estimate median progression-free survival (PFS) and corresponding 95% confidence interval (CI) and survival curves will be plotted.

## Discussion

iRBD is a REM sleep parasomnia, affecting ~1% of the elderly population and compromising quality of life profoundly (1). Given the increasingly well-recognized link between iRBD and α-synuclein-specific neurodegeneration, a strong rationale exists for considering the population of patients with iRBD as ideal for testing possible disease-modifying strategy. Idebenone is a well-described drug that was initially developed against cognitive decline. It is a synthetic quinone with similarities to the naturally occurring CoQ10 (37). Beneficial effects of idebenone have been reported in the clinical trials of some neurodegeneration diseases, including Huntington's disease, Alzheimer's disease, dementia and Friedreich ataxia (38–41). Besides, rare adverse effects of idebenone mainly include allergic reactions, rashes, nausea, loss of appetite, diarrhea, excitement, insomnia, dizziness, etc. with great tolerability and compliance of patients (42). Recent studies revealed that idebenone ameliorated motor dysfunction by regulating mitophagy in MPTP-induced PD mice (21, 22). Other non-motor symptoms such as sleep disorder, autonomic dysfunction, cognitive and psychiatric impairment can also be improved. The underlying mechanisms are multitudinous and comprehensive, including the regulation of 5-hydroxytryptamine neurotransmitter and hippocampal function, the protection against oxidative stress injury, the inhibition of apoptosis signal and the activation of mitochondrial function (20, 43). It seems that a novel indication of idebenone is promising on preventing or delaying neurodegeneration with its favorable safety and tolerability profiles when administrated in a prodromal stage.

In this paper, we outlined our rationale and design of the EITRS study. The EITRS study is a randomized, double-blind, multi-center clinical study is to identify the efficacy and safety of idebenone in the treatment of iRBD into synucleinopathies initially. Three centers participate in the trial and the first patient has been recruited into the study in July 2020. The study is leaded by Ruijin Steering Committee. The Steering Committee members and principal investigators of each center are in charge of the ethics and patient safety. They will have a face-to-face or online meeting to supervise the status and progress of the trial. The diagnosis of iRBD and α-synucleinopathies will be reviewed by independent experts in each center and steering committee. The sponsor of the EITRS trial is the Clinical Research Center, Shanghai Jiao Tong University School of Medicine. The results are expected for publications in 2026.

## Ethics statement

The studies involving human participants were reviewed and approved by Ethics Committees of the Ruijin Hospital affiliated with Shanghai Jiaotong University School of Medicine. The patients/participants provided their written informed consent to participate in this study. Written informed consent was obtained from the individual(s) for the publication of any potentially identifiable images or data included in this article.

## Author contributions

YL, CW, NL, FC, LZ, MN, WK, and JL contributed to conception, design of the study, and collaborate in the implementation of the trial. YL and CW wrote the first draft of the manuscript. NL, FC, LZ, MN, WK, and JL critically revised successive drafts of the paper and approved the final version. All authors contributed to the article and approved the submitted version.

## Funding

This work was supported by grants from the National Natural Science Foundation of China (82071415 and 81873778) and the Innovative Research Team of High-Level Local Universities in Shanghai.

## Conflict of interest

The authors declare that the research was conducted in the absence of any commercial or financial relationships that could be construed as a potential conflict of interest. The reviewer YZ declared a shared parent affiliation with the authors to the handling editor at the time of review.

## Publisher's note

All claims expressed in this article are solely those of the authors and do not necessarily represent those of their affiliated organizations, or those of the publisher, the editors and the reviewers. Any product that may be evaluated in this article, or claim that may be made by its manufacturer, is not guaranteed or endorsed by the publisher.

## Abbreviations

REM: rapid eye movement
RBD: REM sleep behavior disorder
iRBD: idiopathic RBD
PD: Parkinson's disease
DLB: dementia with Lewy bodies
MSA: multiple system atrophy
RSWA: REM sleep atonia loss
18F-FDG PET: ^18^F-flurodeoxyglucose positron emission tomography
1^2^3I-FP-CIT SPECT: ^123^I-N-ω-fluoropropyl-2β-carbomethoxy-3β-(4-iodophenyl) nortropane single-photon emission computed tomography
MRI: magnetic resonance imaging
DAT: dopamine transporter
PSG: polysomnography
AE: serious adverse event
SAE: serious AE
HR: hazard ratio
PFS: progression-free survival
CI: confidence interval
ICSD: International Classification of Sleep Disorders
MDS-UPDRS: Movement Disorder Society-Unified Parkinson's Disease Rating Scale
RBDSQ: REM sleep behavior disorder screening questionnaire
MMSE: Mini-Mental State Examination
MoCA: Montreal Cognitive Assessment
SS-16: Sniffin&#8217 Sticks test-16
HAMD-17: 17-Item Hamilton Rating Scale for Depression
NMSQ: Non-Motor Symptom Questionnaire
SCOPA-AUT: Scales for Outcomes in Parkinson's Disease-Autonomic questionnaire
IL: interleukin
TNF: tumor necrosis factor
CXCL: C-X-C motif ligand
CX3CL1: C-X3-C motif ligand 1
CCL: C-C motif ligand.
